# The genome sequence of a soldier beetle, *Podabrus alpinus* (Paykull, 1798)

**DOI:** 10.12688/wellcomeopenres.18890.1

**Published:** 2023-02-03

**Authors:** Liam M. Crowley, University of Oxford and Wytham Woods Genome Acquisition Lab, Darwin Tree of Life Barcoding collective, Wellcome Sanger Institute Tree of Life programme, Wellcome Sanger Institute Scientific Operations: DNA Pipelines collective, Tree of Life Core Informatics collective, Physilia Chua, Dominik Kusy, Darwin Tree of Life Consortium

**Affiliations:** 1 Department of Biology, University of Oxford, Oxford, Oxfordshire, UK; 2 Wellcome Sanger Institute, Hinxton, Cambridgeshire, UK; 3 Centre of Region Haná for Biotechnological and Agricultural Research, Czech Advanced Technology and Research Institute, Palacký University Olomouc, Olomouc, Czech Republic

**Keywords:** Podabrus alpinus, soldier beetle, genome sequence, chromosomal, Coleoptera

## Abstract

We present a genome assembly from an individual female *Podabrus alpinus* (soldier beetle; Arthropoda; Insecta; Coleoptera; Cantharidae). The genome sequence is 777 megabases in span. Most of the assembly is scaffolded into seven chromosomal pseudomolecules, including the assembled X sex chromosome. The mitochondrial genome has also been assembled and is 18.8 kilobases in length. Gene annotation of this assembly on Ensembl identified 30,955 protein coding genes.

## Species taxonomy

Eukaryota; Metazoa; Ecdysozoa; Arthropoda; Hexapoda; Insecta; Pterygota; Neoptera; Endopterygota; Coleoptera; Polyphaga; Elateriformia; Elateroidea; Cantharidae; Cantharinae; *Podabrus*; *Podabrus alpinus* (Paykull, 1798) (NCBI:txid1553677).

## Background

*Podabrus alpinus* belongs to the Cantharidae family of beetles, also known as soldier beetles. Although quite closely related to click-beetles (Elateridae) ( Kusy *et al.*, 2021), soldier beetles are morphologically very distinct and form phenotypically characteristic lineages along with some other soft-bodied elateroids, i.e., net-winged beetles (Lycidae) and fireflies (Lampyridae), but not with Telegeusidae: Omethinae that had been placed in soldier beetles until quite recently ( Bocakova *et al.*, 2007; Crowson, 1972). The adult *P. alpinus* is medium-sized, elongate, and dorsoventrally depressed with feeble sclerotization of the body, especially the abdomen and elytra. Although highly variable in size and colouration, both light and dark forms are readily recognised among UK Cantharidae by the distinct ‘neck’ and prominent eyes.

*Podabrus alpinus* is widespread and common throughout the northern Palearctic region from western Europe to East Asia ( Kazantsev & Brancucci, 2007). It is also common across much of the UK, except for the east and south-west of England ( NBN Atlas, no date). These beetles are associated with open woodland habitats especially pine trees (Pinaceae), although it is more strongly associated with upland habitats across Europe ( Dvořák, 2010). They are known to the public as they are apparent when sitting on leaves and flowers in forested areas.

Adults are predators, feeding on soft-bodied insects but have also been observed to feed on pollen. Adults are active over a relatively short period between the middle of May to August, and can often be seen resting on umbellifer flowers in warmer weather during the day. In the late afternoons and evenings, they become more active and dispersed. The biology of the larvae is underdocumented, but they are believed to be predators living in upper soil layers and organic debris, in line with other members of the *Podabrus* genus ( Fitton *et al*., 1976).

Most Cantharidae employ chemical defences and aposematic colouration; as a result, Cantharidae are often members of extensive mimicry rings. Therefore, the reference genome of *P. alpinus* could help us understand genomic basis of chemical defence and mimicry. As one of the targeted UK species assembled for the Darwin Tree of Life project, this is the first high-quality genome assembled for *P. alpinus*.

### Genome sequence report

**Figure 1.**
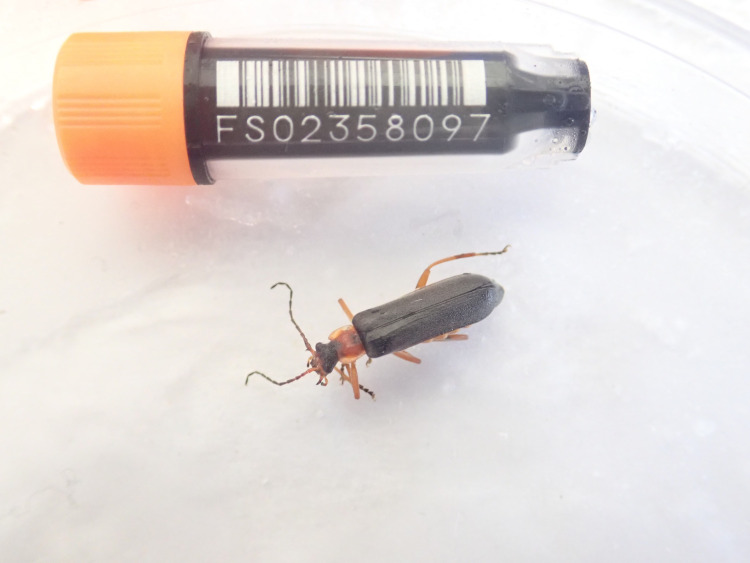
Photograph of the *Podabrus alpinus* (icPodAlpi1) specimen used for genome sequencing.

The genome was sequenced from one female *Podabrus alpinus* specimen ( Figure 1) collected from Wytham Woods (51.768, –1,34). A total of 27-fold coverage in Pacific Biosciences single-molecule HiFi long reads and 43-fold coverage in 10X Genomics read clouds were generated. Primary assembly contigs were scaffolded with chromosome conformation Hi-C data. Manual assembly curation corrected 101 missing joins and misjoins and removed 13 haplotypic duplications, reducing the assembly length by 0.73% and the scaffold number by 10.66%, and increasing the scaffold N50 by 12.32%.

**Figure 2.**
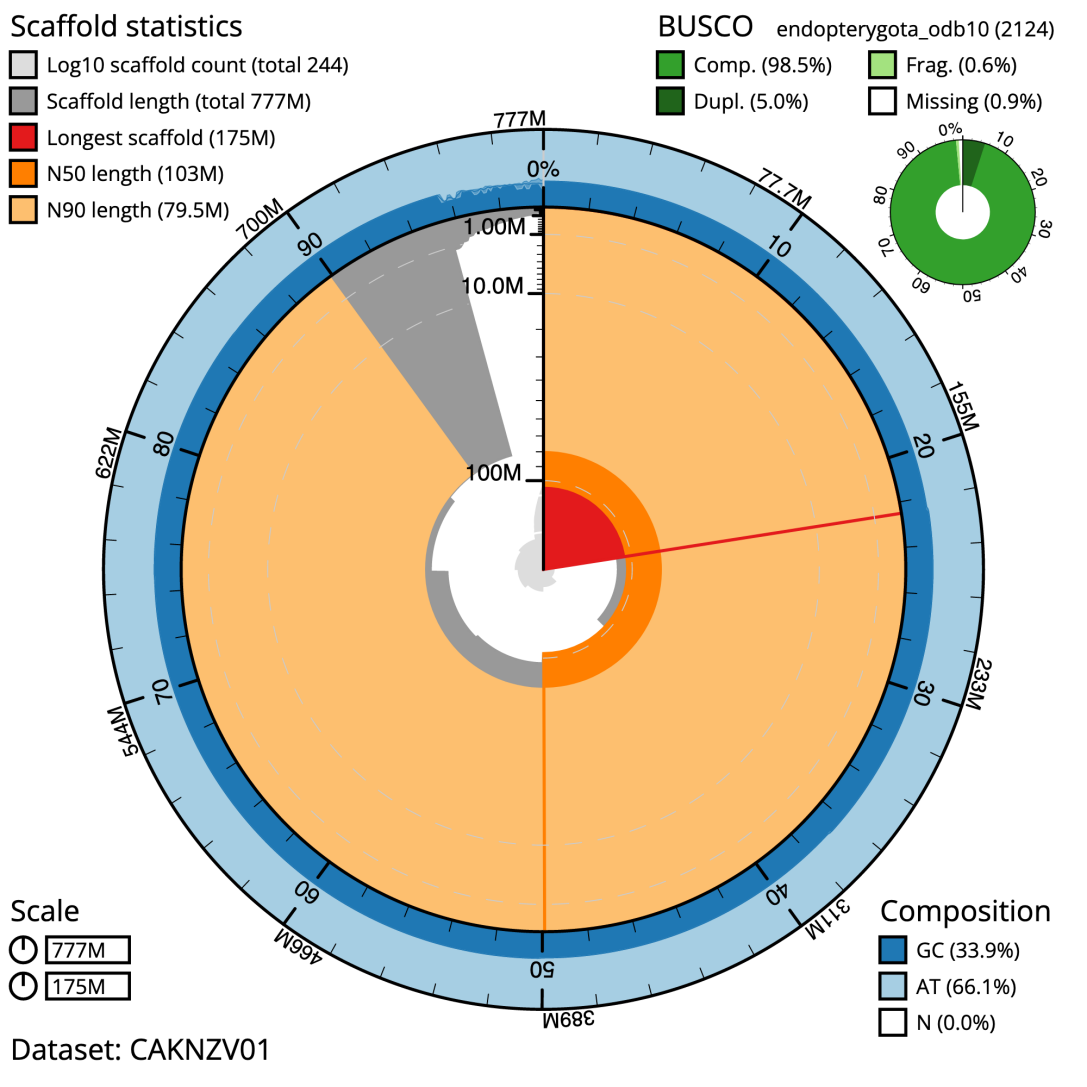
Genome assembly of *Podabrus alpinus*, icPodAlpi1.1: metrics. The BlobToolKit Snailplot shows N50 metrics and BUSCO gene completeness. The main plot is divided into 1,000 size-ordered bins around the circumference with each bin representing 0.1% of the 777,254,275 bp assembly. The distribution of scaffold lengths is shown in dark grey with the plot radius scaled to the longest scaffold present in the assembly (175,353,315 bp, shown in red). Orange and pale-orange arcs show the N50 and N90 scaffold lengths (103,283,173 and 79,473,900 bp), respectively. The pale grey spiral shows the cumulative scaffold count on a log scale with white scale lines showing successive orders of magnitude. The blue and pale-blue area around the outside of the plot shows the distribution of GC, AT and N percentages in the same bins as the inner plot. A summary of complete, fragmented, duplicated and missing BUSCO genes in the endopterygota_odb10 set is shown in the top right. An interactive version of this figure is available at https://blobtoolkit.genomehubs.org/view/icPodAlpi1.1/dataset/CAKNZV01/snail.

The final assembly has a total length of 777.2 Mb in 243 sequence scaffolds with a scaffold N50 of 103.3 Mb ( Table 1). Most (95.62%) of the assembly sequence was assigned to seven chromosomal-level scaffolds, representing six autosomes and the X sex chromosome. Chromosome-scale scaffolds confirmed by the Hi-C data are named in order of size ( Figure 2– Figure 5; Table 2). The orientation of chromosome 2 in the region 5.5–10.6 Mb is uncertain. The assembly has a BUSCO v5.3.2 ( Manni *et al.*, 2021) completeness of 98.5% (single 93.5%, duplicated 5%) using the endopterygota_odb10 reference set ( *n* = 2,124). While not fully phased, the assembly deposited is of one haplotype. Contigs corresponding to the second haplotype have also been deposited.

**Table 1.** Genome data for *Podabrus alpinus*, icPodAlpi1.1.

Project accession data	
Assembly identifier	icPodAlpi1.1
Species	*Podabrus alpinus*
Specimen	icPodAlpi1
NCBI taxonomy ID	1553677
BioProject	PRJEB49177
BioSample ID	SAMEA7520644
Isolate information	female, whole organism
Raw data accessions	
PacificBiosciences SEQUEL II	ERR7756498
10X Genomics Illumina	ERR7569937–ERR7569940
Hi-C Illumina	ERR7569941
Genome assembly	
Assembly accession	GCA_932274525.1
*Accession of alternate haplotype*	GCA_932273715.1
Span (Mb)	777.3
Number of contigs	391
Contig N50 length (Mb)	9.9
Number of scaffolds	243
Scaffold N50 length (Mb)	103.3
Longest scaffold (Mb)	175.4
BUSCO *	C:98.5%[S:93.5%,D:5.0%], F:0.6%,M:0.9%,n:2,124
Genome annotation	
Number of protein-coding genes	30,955
Number of gene transcripts	31,403

* BUSCO scores based on the endopterygota_odb10 BUSCO set using v5.3.2. C = complete [S = single copy, D = duplicated], F = fragmented, M = missing, n = number of orthologues in comparison. A full set of BUSCO scores is available at https://blobtoolkit.genomehubs.org/view/icPodAlpi1.1/dataset/CAKNZV01/busco.

**Figure 3.**
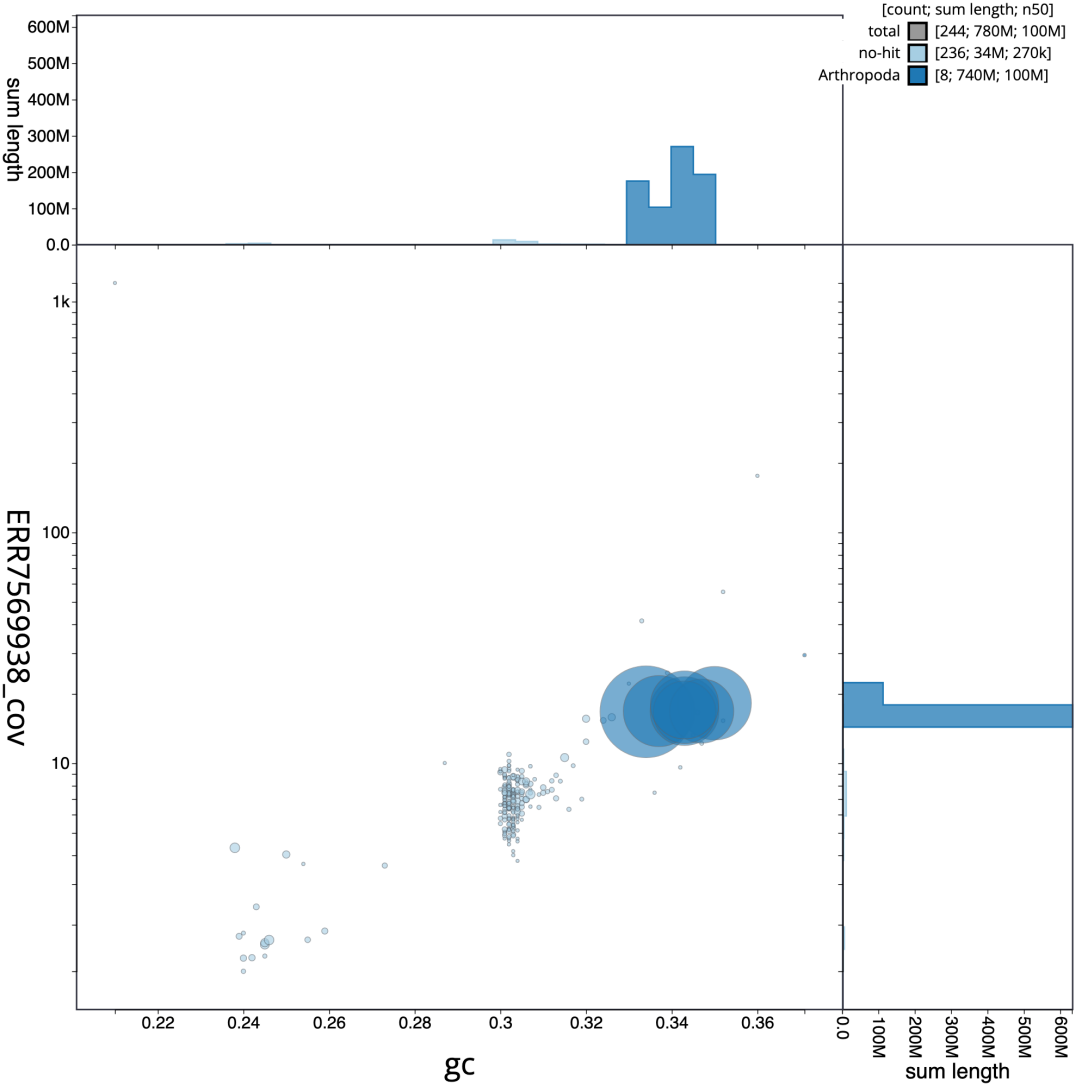
Genome assembly of *Podabrus alpinus*, icPodAlpi1.1: GC coverage. BlobToolKit GC-coverage plot. Scaffolds are coloured by phylum. Circles are sized in proportion to scaffold length. Histograms show the distribution of scaffold length sum along each axis. An interactive version of this figure is available at https://blobtoolkit.genomehubs.org/view/icPodAlpi1.1/dataset/CAKNZV01/blob.

**Figure 4.**
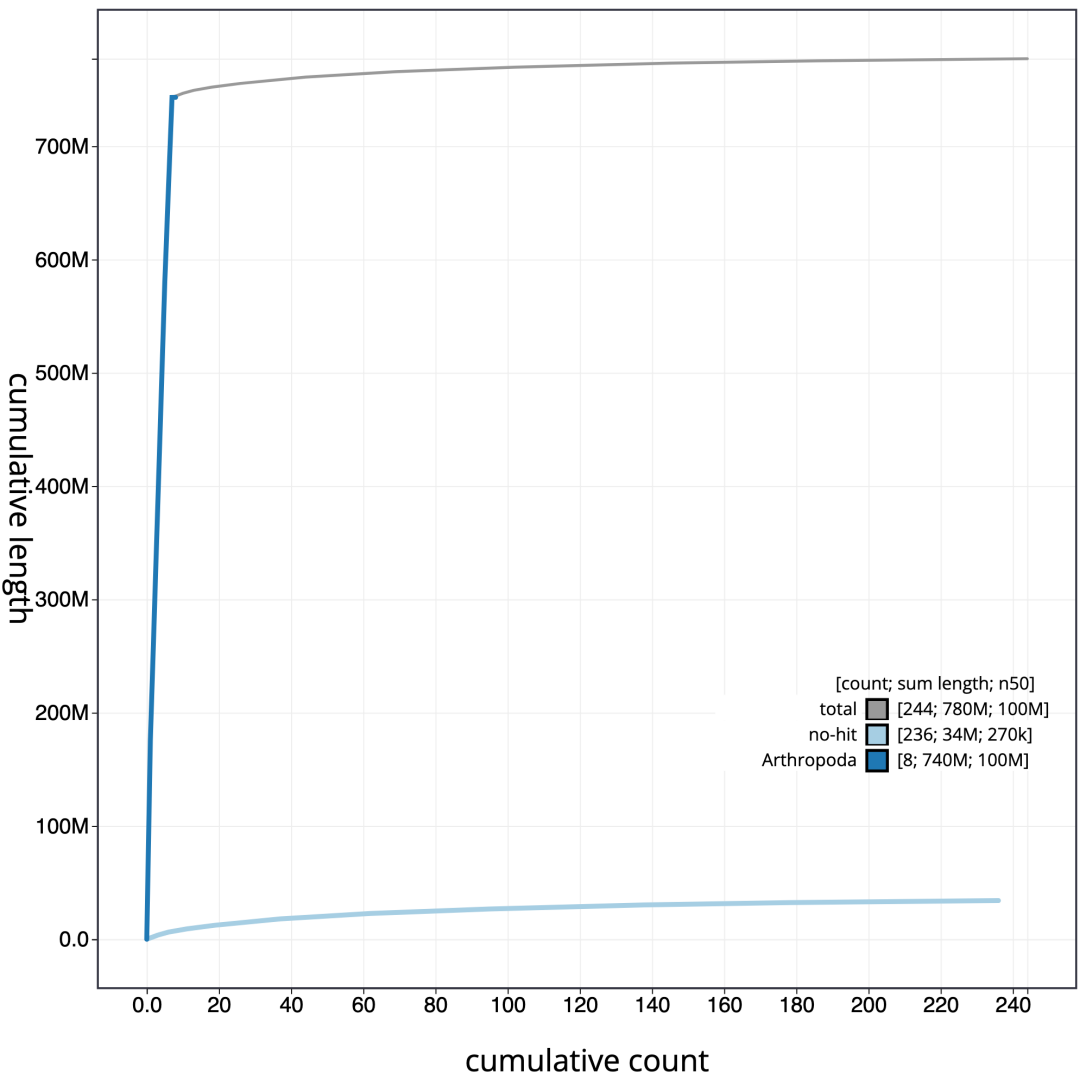
Genome assembly of *Podabrus alpinus*, icPodAlpi1.1: cumulative sequence. BlobToolKit cumulative sequence plot. The grey line shows cumulative length for all scaffolds. Coloured lines show cumulative lengths of scaffolds assigned to each phylum using the buscogenes taxrule. An interactive version of this figure is available at https://blobtoolkit.genomehubs.org/view/icPodAlpi1.1/dataset/CAKNZV01/cumulative.

**Figure 5.**
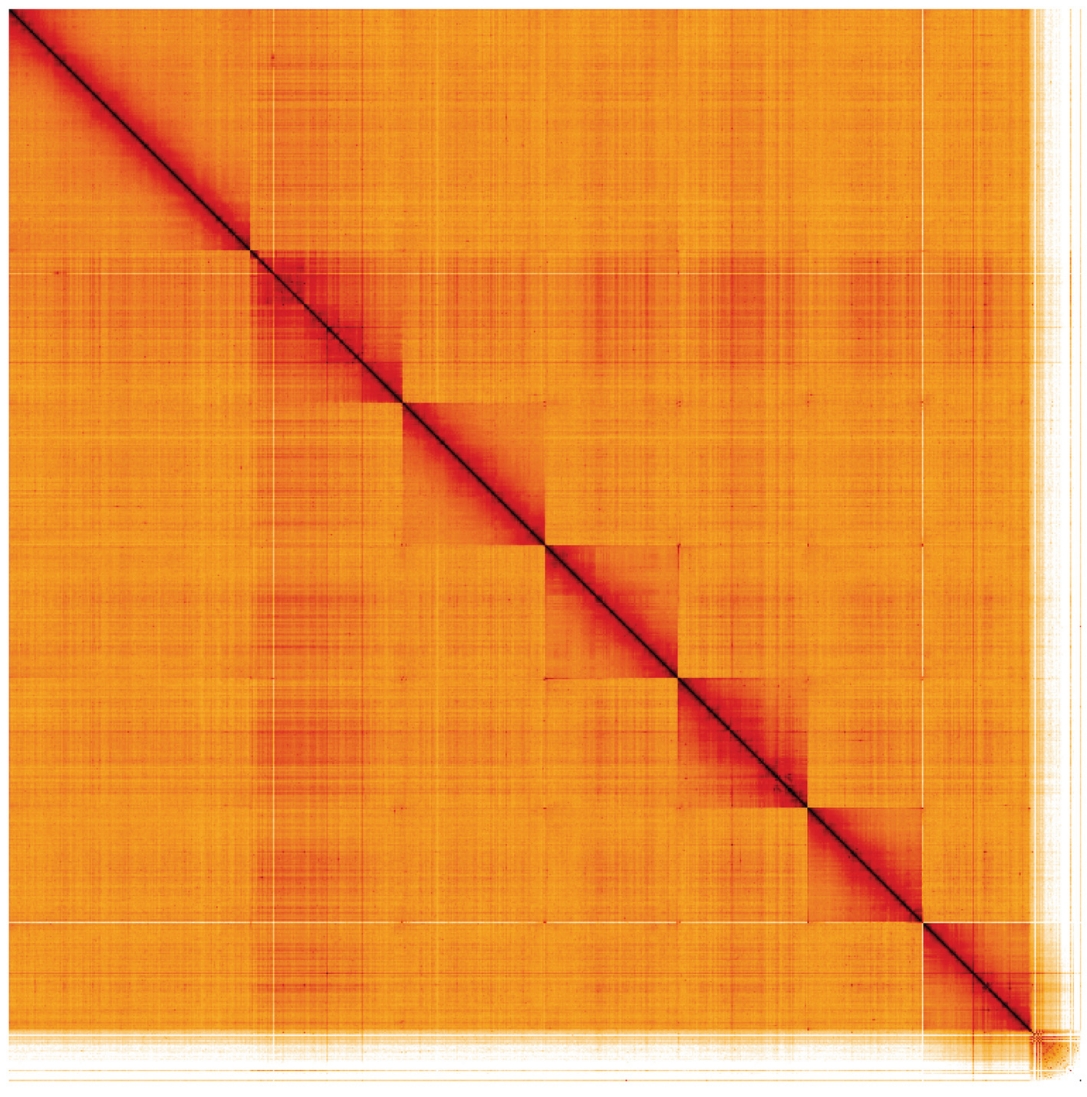
Genome assembly of *Podabrus alpinus*, icPodAlpi1.1: Hi-C contact map. Hi-C contact map of the icPodAlpi1.1 assembly, visualised using HiGlass. Chromosomes are shown in order of size from left to right and top to bottom. An interactive version of this figure may be viewed at https://genome-note-higlass.tol.sanger.ac.uk/l/?d=X14WXTTaQ9el1HLkgj9fMw.

**Table 2.** Chromosomal pseudomolecules in the genome assembly of *Podabrus alpinus*, icPodAlpi1.

INSDC accession	Name	Size (Mb)	GC%
OW026451.1	1	175.35	33.4
OW026452.1	2	110.77	35
OW026453.1	3	103.28	33.7
OW026454.1	4	96.26	34.3
OW026456.1	5	83.25	34.7
OW026457.1	6	79.47	34.3
OW026455.1	X	94.67	34.3
OW026458.1	MT	0.02	21
-	unplaced	34.17	29.3

### Genome annotation report

The *P. alpinus* genome assembly (cPodAlpi1.1) was annotated using the Ensembl rapid annotation pipeline ( Table 1; https://rapid.ensembl.org/Podabrus_alpinus_GCA_932274525.1/). The resulting annotation includes 31,403 transcribed mRNAs from 30,955 protein-coding genes.

## Methods

### Sample acquisition and nucleic acid extraction

A female *P. alpinus* (icPodAlpi1) was collected from Wytham Woods, Oxfordshire (biological vice-county: Berkshire), UK (latitude 51.77, longitude –1.34) by Liam Crowley (University of Oxford) on 22 May 2020. The sample was taken from woodland by netting and preserved on dry ice.

DNA was extracted at the Tree of Life laboratory, Wellcome Sanger Institute (WSI). The icPodAlpi1 sample was weighed and dissected on dry ice with tissue set aside for Hi-C sequencing. Whole body tissue was disrupted using a Nippi Powermasher fitted with a BioMasher pestle. High molecular weight (HMW) DNA was extracted using the Qiagen MagAttract HMW DNA extraction kit. Low molecular weight DNA was removed from a 20 ng aliquot of extracted DNA using 0.8X AMpure XP purification kit prior to 10X Chromium sequencing; a minimum of 50 ng DNA was submitted for 10X sequencing. HMW DNA was sheared into an average fragment size of 12–20 kb in a Megaruptor 3 system with speed setting 30. Sheared DNA was purified by solid-phase reversible immobilisation using AMPure PB beads with a 1.8X ratio of beads to sample to remove the shorter fragments and concentrate the DNA sample. The concentration of the sheared and purified DNA was assessed using a Nanodrop spectrophotometer and Qubit Fluorometer and Qubit dsDNA High Sensitivity Assay kit. Fragment size distribution was evaluated by running the sample on the FemtoPulse system.

### Sequencing

Pacific Biosciences HiFi circular consensus and 10X Genomics read cloud DNA sequencing libraries were constructed according to the manufacturers’ instructions. DNA sequencing was performed by the Scientific Operations core at the WSI on Pacific Biosciences SEQUEL II (HiFi) and HiSeq X Ten (10X) instruments. Hi-C data were also generated from tissue of icPodAlpi1 using the Arima v2 kit and sequenced on the HiSeq X Ten instrument.

### Genome assembly

Assembly was carried out with Hifiasm ( Cheng *et al.*, 2021) and haplotypic duplication was identified and removed with purge_dups ( Guan *et al.*, 2020). One round of polishing was performed by aligning 10X Genomics read data to the assembly with Long Ranger ALIGN, calling variants with freebayes ( Garrison & Marth, 2012). The assembly was then scaffolded with Hi-C data ( Rao *et al.*, 2014) using YaHS ( Zhou *et al.*, 2022). The assembly was checked for contamination as described previously ( Howe *et al.*, 2021). Manual curation was performed using HiGlass ( Kerpedjiev *et al.*, 2018) and Pretext ( Harry, 2022). The mitochondrial genome was assembled using MitoHiFi ( Uliano-Silva *et al.*, 2022), which performed annotation using MitoFinder ( Allio *et al.*, 2020). The genome was analysed and BUSCO scores generated within the BlobToolKit environment ( Challis *et al.*, 2020). Table 3 contains a list of all software tool versions used, where appropriate.

**Table 3.** Software tools and versions used.

Software tool	Version	Source
BlobToolKit	3.2.6	Challis *et al.*, 2020
freebayes	1.3.1-17- gaa2ace8	Garrison & Marth, 2012
Hifiasm	0.15.3	Cheng *et al.*, 2021
HiGlass	1.11.6	Kerpedjiev *et al.*, 2018
Long Ranger ALIGN	2.2.2	https://support.10xgenomics.com/ genome-exome/software/pipelines/ latest/advanced/other-pipelines
MitoHiFi	2	Uliano-Silva *et al.*, 2022
PretextView	0.2	Harry, 2022
purge_dups	1.2.3	Guan *et al.*, 2020
YaHS	1	Zhou *et al.* 2022

### Genome annotation

The BRAKER2 pipeline ( Brůna *et al.*, 2021) was used in the default protein mode to generate annotation for the *Podabrus alpinus* assembly (GCA_932274525.1) in Ensembl Rapid Release.

## Data Availability

European Nucleotide Archive: *Podabrus alpinus*. Accession number PRJEB49177; https://identifiers.org/ena.embl/PRJEB49177 ( Wellcome Sanger Institute, 2021) The genome sequence is released openly for reuse. The *P. alpinus* genome sequencing initiative is part of the Darwin Tree of Life (DToL) project. All raw sequence data and the assembly have been deposited in INSDC databases. Raw data and assembly accession identifiers are reported in Table 1.

## Author information

Members of the University of Oxford and Wytham Woods Genome Acquisition Lab are listed here: https://doi.org/10.5281/zenodo.4789929.

Members of the Darwin Tree of Life Barcoding collective are listed here: https://doi.org/10.5281/zenodo.4893703.

Members of the Wellcome Sanger Institute Tree of Life programme are listed here: https://doi.org/10.5281/zenodo.4783585.

Members of Wellcome Sanger Institute Scientific Operations: DNA Pipelines collective are listed here: https://doi.org/10.5281/zenodo.4790455.

Members of the Tree of Life Core Informatics collective are listed here: https://doi.org/10.5281/zenodo.5013541.

Members of the Darwin Tree of Life Consortium are listed here: https://doi.org/10.5281/zenodo.4783558.
