# Supplementary material for: The genome sequence of a soldier beetle, Podabrus alpinus (Paykull, 1798)
Source: Wellcome Open Res. 2023 Feb 3;8:56. [Version 1] doi: 10.12688/wellcomeopenres.18890.1 (PMC10280036; doi:10.12688/wellcomeopenres.18890.1)
Supplement: Peer review history

# Peer review: Reviewer response for version 1

**DOI:** 10.21956/wellcomeopenres.20946.r58668

**Published:** 2023-06-22

**Authors:** Luciano Palmieri

**Affiliations:** 1 Department of Integrative Biology, University of Wisconsin-Madison, Madison, Wisconsin, USA

The article presents the genome assembly of a female *Podabrus alpinus* (soldier beetle) and provides information on its characteristics and biological significance. The rationale for creating the dataset is explicitly described and the authors mention that the reference genome could aid in understanding the genomic basis of chemical defense and mimicry in the Cantharidae family. The protocols used for sequencing and assembly, including PacBio HiFi long reads and 10X short reads, along with chromosome conformation Hi-C data, are considered appropriate. The methods section provides sufficient details on sample acquisition, nucleic acid extraction, sequencing, assembly, and annotation. The article provides sufficient details to allow replication by others.

 The work is technically sound as it involves manual assembly curation and removal of haplotypic duplications. The genome assembly is close to 770 megabases in length and is scaffolded into seven chromosomal pseudomolecules, including the X sex chromosome, and the mitochondrial genome. The assembly is reported to have a high completeness score. Gene annotation was performed using the Ensembl pipeline, resulting in the identification of 30,955 protein-coding genes. The article is well written and concise, using appropriate terminology, and I recommend it for publication in Open Science as it is.

 One small detail I would suggest fixing is the way the geographical coordinates are presented in the “Genome Sequence Report” section, second line. It would be better to present it as in the “Sample acquisition and nucleic acid extraction” section, also second line, or remove it, as the coordinates are presented later in the manuscript.

Are sufficient details of methods and materials provided to allow replication by others?

Yes

Is the rationale for creating the dataset(s) clearly described?

Yes

Are the datasets clearly presented in a useable and accessible format?

Yes

Are the protocols appropriate and is the work technically sound?

Yes

Reviewer Expertise:

Phylogenomics, Systematics, Insect taxonomy, and Macroevolution.

I confirm that I have read this submission and believe that I have an appropriate level of expertise to confirm that it is of an acceptable scientific standard.

# Peer review: Reviewer response for version 1

**DOI:** 10.21956/wellcomeopenres.20946.r58664

**Published:** 2023-06-22

**Authors:** Nagdy F Abdel-Baky

**Affiliations:** 1 Department of Plant Production and Protection, College of Agriculture and Veterinary Medicine, Qassim University, Buraydah, Al Qassim, Saudi Arabia

The data note report in the title: The genome sequence of a soldier beetle, ***Podabrus alpinus*** (Paykull, 1798) by Crowley et al., describes the genome sequencing, assembly, and annotation from an individual female *Podabrus alpinus* (soldier beetle; Arthropoda; Insecta; Coleoptera; Cantharidae).

 The full genome size contained 777 Mbs, the Number of contigs counted 391, the number of protein-coding genes (30,955), and the number of gene transcripts (31,403). Furthermore, the mtDNA reached 18.8 Kbs in length.

 The report is well-designed and written, and the concept is clear.

Are sufficient details of methods and materials provided to allow replication by others?

Yes

Is the rationale for creating the dataset(s) clearly described?

Yes

Are the datasets clearly presented in a useable and accessible format?

Yes

Are the protocols appropriate and is the work technically sound?

Yes

Reviewer Expertise:

Entomology, IPM, Insect genetics

I confirm that I have read this submission and believe that I have an appropriate level of expertise to confirm that it is of an acceptable scientific standard.

# Peer review: Reviewer response for version 1

**DOI:** 10.21956/wellcomeopenres.20946.r58671

**Published:** 2023-06-19

**Authors:** Fahad H Alqahtani

**Affiliations:** 1 National Center for Agricultural Technology, National Center for Agricultural Technology, King Abdulaziz City for Science and Technology, Riyadh, Saudi Arabia

The authors yielded a high completeness genome assembly at a chromosome level and high-quality annotation for a female soldier beetle, Podabrus alpinus (Paykull, 1798). The genome assembly was performed using three sequencing technologies: PacificBiosciences SEQUEL II, 10X Genomics Illumina, and Hi-C Illumina. The completeness of genome assembly was determined using BUSCO analysis which yielded an N50 scaffold length of 103.3 Mb and a 98.5% of common genes were completely present. However, minor comments should be addressed: 

- I suggest including a phylogeny analysis of the mitochondrial genome, which might help maximize the impact of the work.
- Regarding the mitochondrial genome annotation, the Mitofinder tool uses closely related species to guide the annotation. I would then suggest mentioning this species in the method section.
- "Pretext" in the Genome assembly section should be  "PretextView".
- I suggest adding the number of scaffolds assigned for each chromosome to the table 2. 

Are sufficient details of methods and materials provided to allow replication by others?

Yes

Is the rationale for creating the dataset(s) clearly described?

Yes

Are the datasets clearly presented in a useable and accessible format?

Yes

Are the protocols appropriate and is the work technically sound?

Yes

Reviewer Expertise:

Interested in bioinformatics, with an emphasis on mitochondrial DNA analysis.

I confirm that I have read this submission and believe that I have an appropriate level of expertise to confirm that it is of an acceptable scientific standard.

# Peer review: Reviewer response for version 1

**DOI:** 10.21956/wellcomeopenres.20946.r54741

**Published:** 2023-02-07

**Authors:** Robert B. Angus

**Affiliations:** 1 Department of Life Sciences (Insects), The Natural History Museum, London, UK

This is a clearly presented piece of work and appears to be completely sound, though I confess I am not able to assess detailed DNA protocols. The organisation of the genome into units representing 6 pairs of autosomes plus the X chromosome gives a karyotype corresponding with those of various *Cantharis* and *Rhagonycha* species reported by L. V. James & R. B. Angus,  2007  A chromosomal investigation of some British Cantharidae (Coleoptera).  Genetica 130 (3): 293 - 300.

Are sufficient details of methods and materials provided to allow replication by others?

Yes

Is the rationale for creating the dataset(s) clearly described?

Yes

Are the datasets clearly presented in a useable and accessible format?

Yes

Are the protocols appropriate and is the work technically sound?

Yes

Reviewer Expertise:

Taxonomy, including cytotaxonomy of Coleoptera

I confirm that I have read this submission and believe that I have an appropriate level of expertise to confirm that it is of an acceptable scientific standard.

## References

[1] : A chromosomal investigation of some British Cantharidae (Coleoptera). *Genetica* .2007;130(3) : 10.1007/s10709-006-9106-5 293-300 10.1007/s10709-006-9106-5 17004007
